# Molecular regulation and clinical significance of caveolin‐1 methylation in chronic lung diseases

**DOI:** 10.1002/ctm2.2

**Published:** 2020-04-16

**Authors:** Furong Yan, Lili Su, Xiaoyang Chen, Xiangdong Wang, Hongzhi Gao, Yiming Zeng

**Affiliations:** ^1^ Clinical Center for Molecular Diagnosis and Therapy Second Affiliated Hospital of Fujian Medical University Quanzhou Fujian China; ^2^ Department of Pulmonary and Critical Care Medicine Respiratory Medicine Center of Fujian Province Second Affiliated Hospital of Fujian Medical University Quanzhou Fujian China

**Keywords:** caveolae, caveolin‐1, chronic lung diseases, epigenetic modification, methylation

## Abstract

Chronic lung diseases represent a largely global burden whose pathogenesis remains largely unknown. Research increasingly suggests that epigenetic modifications, especially DNA methylation, play a mechanistic role in chronic lung diseases. DNA methylation can affect gene expression and induce various diseases. Of the caveolae in plasma membrane of cell, caveolin‐1 (Cav‐1) is a crucial structural constituent involved in many important life activities. With the increasingly advanced progress of genome‐wide methylation sequencing technologies, the important impact of Cav‐1 DNA methylation has been discovered. The present review overviews the biological characters, functions, and structure of Cav‐1; epigenetic modifications of Cav‐1 in health and disease; expression and regulation of Cav‐1 DNA methylation in the respiratory system and its significance; as well as clinical potential as disease‐specific biomarker and targets for early diagnosis and therapy.

**Abbreviations**
COPD: chronic obstructive pulmonary disease
CSD: caveolin scaffolding domain
EGF: epidermal growth factor
eNOS: endothelial nitric oxide synthase
IL‐6: interleukin‐6
IPF: idiopathic pulmonary fibrosis
LC3B: light chain 3B
MAP: mitogen‐activated protein
PDGF: platelet‐derived growth factor
TGF‐β1: transforming growth factor‐β1

## 1 BACKGROUND

**FIGURE 1:**
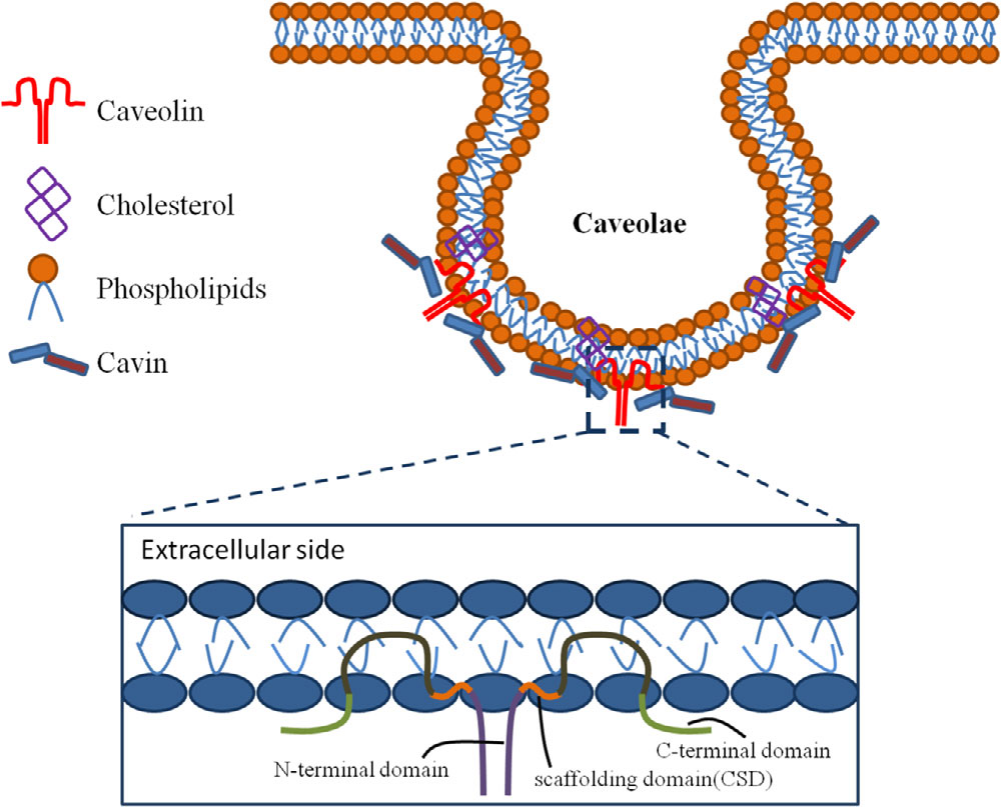
The structure of caveolae. Caveolae are a kind of flask‐shaped invaginations in the plasma membrane, including caveolin, cavin (also named polymerase I and transcript release factor (PTRF)), lipids, and so on. Cav‐1 is the major integral membrane protein for the assembly of caveolae. Cav‐1 contains a highly conserved domain named caveolin scaffolding domain (CSD, acids 82‐101). The CSD domain takes part in the interactions with signaling proteins and regulates signal transduction in various cellular processes

Caveolae are crucial in various cellular, physiological, and pathological processes, for example, cell proliferation, apoptosis, migration, differentiation, angiogenesis, tumorigenesis, and metastasis by special signal transduction, endocytosis, and transcytosis. Caveolae are a kind of flask‐like invaginations in the plasma membrane. The constitute of caveolae includes caveolin, cavin (also named polymerase I and transcript release factor), lipids, transcription polymerase, as well as various ion channel proteins (Figure 1).^1^ The caveolin family contains three subtypes: caveolin‐1 (Cav‐1), caveolin‐2 (Cav‐2), and caveolin‐3 (Cav‐3), of which Cav‐1 is co‐expressed primarily in many cells with Cav‐2. Cav‐2 is not essential in the formation of caveolae and can be located or expresses dependently on Cav‐1.^2^ Cav‐3 is only specific to muscle cells.^3^ Cav‐1 is the major integral membrane protein for the assembly of caveolae in nonmuscle cells. Emerging evidence demonstrates that Cav‐1 plays a positive or negative regulatory role in cell signaling transduction, which depends on the type of cells and signaling pathways. The changes in expression of Cav‐1 may be vital on chronic lung diseases as a new target for the treatment. Our review aims at overviewing the biological characters, structure and function of Cav‐1, epigenetic modifications of Cav‐1 in health and disease, expression and regulation of Cav‐1 in the respiratory system, Cav‐1 methylation and significance in chronic lung diseases, as well as clinical potential as disease‐specific biomarker and targets for early diagnosis and therapy.

### 1.1 Structure and functions of Cav‐1

Cav‐1 consists of 178 amino acid residues with a highly conserved amphipathic region of caveolin scaffolding domain (CSD), which interacts with signaling proteins and regulates signal transduction through the entrapment of signaling partners.^4^ Cav‐1 monomers may form a disk‐shaped oligomer with its carboxyl terminal part toward the center and insert into the plasma membrane by CSD and intramembrane domain, a second amphipathic helix.^5^ CSD is dynamically allocated between fully helical or partly unstructured forms, which determine its accessibility.^6^ The structure of Cav‐1 is decided by its oligomerization state and the organization of other components in the caveolae like cavin or lipids. In caveolae, Cav‐1 oligomers converge specific lipids such as cholesterol, phosphatidylinositol‐4,5‐bisphosphate, and phosphatidyl serine to aggregate cavin trimers. Caveolae are enriched in multiple lipids, some of which are highly important signaling molecules in cell membrane.^7^ Cav‐1 closely connects with cholesterol and sphingolipids. This connection cannot be separated at low temperatures by high concentrations of salts or nonionic surfactant detergents likes triton X‐100.^8^ Cav‐1 can interact with cholesterol at a 1:1 stoichiometry through a sequence that matches to cholesterol recognition/interaction amino acid consensus domain.^9^ Depletion of cholesterol causes a decrease in caveolae. Conversely, cholesterol supplementation will increase membrane cholesterol, and lead to a decrease in membrane fluidity and an increase in caveolae and Cav‐1 number on the cell membrane.^10^

Cav‐1 is encoded on 7q31.1 of human chromosome and critical in the formation of caveolae. Caveolae cannot be formed without Cav‐1. Genetic deletion of Cav‐1 caused the lack of caveolae.^11^, ^12^ The amino‐ and carboxy‐terminal domains of Cav‐1 are limited in the cytoplasmic surface of cell membrane and long putative hairpin intramembrane domain.^13^ Cav‐1 exists into two isoforms: Cav‐1α and Cav‐1β, having similar structures to CSD and an acetylated C‐terminus.^14^ The only difference between Cav‐1α and Cav‐1β structures is that the Cav‐1α has an N‐terminal 31 amino acids rather than Cav‐1β.^15^ Cav‐1α and Cav‐1β are produced from two distinct mRNAs. Full‐length mRNA may produce the Cav‐1α predominantly, but little Cav‐1β. The Cav‐1β was most partly generated from 5'‐end variant mRNA.^16^ The function of Cav‐1 isoforms differs, for example, Cav‐1α primarily expressed as an early marker for vasculogenesis during the development of lung blood vessels and in alveolar Type I cells in mature lungs.^17^ Hyperexpression of Cav‐1β may inhibit activation of the bone morphogenetic proteins pathway signaling, rather than Cav‐1α.^14^, ^18^ In freeze‐fracture immunoelectron microscopy, the α/β ratio in human fibroblasts is higher in the deep of caveolae than the shallow ones. The different ratio of Cav‐1 isoforms in the deep and shallow of caveolae shows a unique molecular mechanism about the caveolae‐shaped differentiation.^19^

Phosphatidylserine was accumulated on the cytoplasmic surface of the plasma membrane related to caveolae and the function of Cav‐1.^20^ Cav‐1 is phosphorylated on tyrosine‐14 in response to stimulation, responsible for various biological processes covering signal transduction and regulation in caveolae.^21^ The phosphorylation of Cav‐1 at Tyr14 can be regulated in posttranslational level to contribute to the pathogenesis of lung diseases.^22^ Changes of the phosphorylation of Cav‐1 may be the direction of targeted therapy. Phosphorylation of Cav‐1 is a necessary process to enhance the interaction with endothelial nitric oxide synthase (eNOS)^23^ and regulate nanoclustering of isotype‐specific B‐cell antigen receptors.^24^ Curcumin prevented kidney injury in diabetic nephropathy by inhibiting phosphorylation of Cav‐1.^25^ Lipopolysaccharide (LPS)‐induced phosphorylation of Cav‐1‐enhanced microvascular permeability.^26^ Various important processes are affected by Cav‐1. Cav‐1 gathering with other signal‐sensing molecules can be activated by appropriate stimulation. Many growth factors, signaling receptors, kinases, enzymes, and other signaling regulators are clustered in caveolae.^27^ Cav‐1 can interact with regulatory factors of signaling pathways, such as Akt,^28^ Src kinases,^29^ Rab5 small GTPases,^30^ and is also involved in maintenance of the immune system. Cav‐1 is closely associated with eNOS, which is mainly reflected in the co‐localization and the dynamic functional regulation.^31^ In endothelial cells, eNOS directly binds with the CSD of Cav‐1 and co‐expressed with it at a special ratio.^23^ On the one hand, Cav‐1 regulates eNOS expression level and inhibits its activity when activated, and, on the other hand, sustained eNOS‐derived NO production leads to the degradation of Cav‐1.^31^ The association between Cav‐1 and eNOS was crucial in vascular homeostasis when confronted with oxidative stress, which was found in several disease states including atherosclerosis, diabetes, and myocardial infarction.^32^, ^33^ Epidermal growth factor (EGF) and platelet‐derived growth factor (PDGF) receptors were transiently associated with Cav‐1 in the presence of ligand. Overexpressed Cav‐1 suppressed p42/44 mitogen‐activated protein (MAP) kinase activation and cell proliferation induced by EGF and PDGF.^34^, ^35^ The p42/44 MAP kinase could be activated in the absence of Cav‐1, leading to cardiac hypertrophy.^36^ Cav‐1 regulates the distribution of nanoclusters of isotype‐specific B‐cell antigen receptors in B cell plasma membrane, a multiprotein complex, which plays an important role in the development, proliferation, and activation of B cells, to prevent B‐cell‐induced autoimmunity.^24^ Cav‐1 contributes to mitochondrial fatty acid catabolism and respiration through modulating mitochondrial cholesterol levels, stimulating peroxisome proliferator‐activated receptor α–dependent fatty acid oxidation and enhancing ketogenesis production.^37^, ^38^ Cav‐1 affects the regulation of glycolytic activities. Isoflurane inhibits cell apoptosis through increasing glycolysis in a Cav‐1‐dependent mechanism.^39^ Cav‐1 also plays part roles in the regulation of apoptosis. Microtubule‐associated protein 1 light chain 3B (LC3B), as a vital regulator of autophagic and apoptotic signaling cascades, requires Cav‐1 to form a complex with the death receptor Fas to regulate apoptosis.^40^ The absence of Cav‐1 caused various disorders related to normal life activities, leading to diseases.^41^ Cav‐1 knockout mice have multiple functional disorders including hyperglycaemia, lipidosis, and dysfunction in vascular permeability.^12^, ^42^, ^43^, ^44^ Cav‐1 mutation leads to severely lipodystrophic diabetes.^45^ Caveolae as the multifunctional organelle is important for the regulation of various cellular functions. Understanding of caveolae is useful to design therapies for caveolae‐associated diseases

### 1.2 Cav‐1 in the respiratory system

**FIGURE 2:**
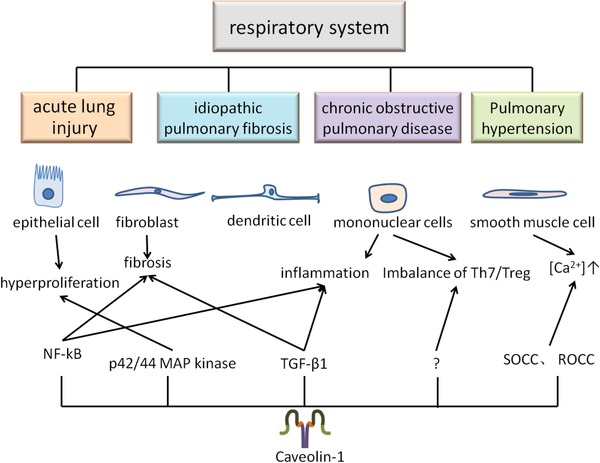
Cav‐1 in the respiratory system. Caveolae widely exists in many cells of the respiratory system and are involved in various cellular activities. Cav‐1 as the important structural proteins of caveolae is involved in a variety of signaling pathways, and the abnormal expression level of it will lead to the structural and functional dysfunction and induce the occurrence of diseases

Caveolae and their vital constituent Cav‐1 play complex and significant roles in respiratory system. Within the lung, caveolae are widely present in airway or alveolar epithelium, airway or pulmonary artery smooth muscle, pulmonary endothelium, fibroblasts, and immune cells.^46^ Thus, the widespread presence of caveolae raises the controllability of themselves and Cav‐1 in lung disease states and can in turn influence the pathophysiology. The changes in Cav‐1 expression lead to a series of function and morphological dysfunction in the respiratory system (Figure 2).

Cav‐1 has a range of functions and effects, many of which are harmful, but some may also promote health. In addition, Cav‐1 is found in a variety of cells and has different roles in these cell types. Therefore, it needs to be studied separately in cell culture and expression analysis and animal disease models.^47^ The regulation of Cav‐1 is multifunctional in chronic lung diseases. In most of the lung diseases, the expression of Cav‐1 is lower compared to normal conditions. Complete loss of caveolae and Cav‐1 in airways and vasculature is thought to occur in inflammatory lung diseases such as chronic obstructive pulmonary disease (COPD), asthma, and inflammation‐induced lung injury.^48^ Downregulation of Cav‐1 may be related to pulmonary fibrosis due to increased extracellular matrix production, hypercellularity, inflammation, and dysfunction of epithelial barrier.^49^ Cav‐1‐knockout mice enhanced the severity of transforming growth factor‐β1 (TGF‐β1)‐induced oxidative stress, inflammation, and fibrosis.^50^ Deletion of Cav‐1 in mice also developed pulmonary hypertension, myocardial hypertrophy, and alveolar cell hyperproliferation through the activation of p42/44 MAP kinases.^51^ Cav‐1 may regulate pulmonary vascular homeostasis through influencing endothelial angiotensin‐1 converting enzyme expression and activity, of which reduced expression of Cav‐1 leads to abnormal pulmonary vascular development.^52^ COPD is a type of emphysema and/or chronic bronchitis characterized by airflow obstruction. Chronic bronchitis is inflammation that occurs on the inner wall of airway. Emphysema is related to the destruction of the alveoli cells. In addition, oxidative stress, apoptosis, and aging are all involved in COPD. Cav‐1 regulates these processes. For example, loss of Cav‐1 is related to the deficiency of elastic fibers in the lung from the damaged parenchyma of COPD patients.^53^ The expression of Cav‐1 is required in lung fibroblasts and emphysema aging induced by smoking.^54^ The imbalance of Th17/Treg cells was crucial in the pathogenesis of COPD. Cav‐1 is related to the homeostasis of Th17/Treg cells in respiratory inflammation.^55^ Downregulation of Cav‐1 was accompanied by an increase in Treg and decrease in Th17 expression. These results indicate that Cav‐1 plays a pivotal role in the occurrence and development of COPD. Cav‐1 was found to be involved in the formation of pulmonary capillary leakage, pulmonary edema, and lung injury during acute inflammatory response.^56^, ^57^ Cav‐1 deletion enhanced expression of the pro‐inflammatory cytokines stimulated by LPS. The expression of Cav‐1 was downregulated in peripheral monocytes or plasma harvested from patients with asthma or COPD along with pulmonary hypertension.^58^, ^59^ Fibrotic disorders are related to the abnormal accumulation of fibroblasts in tissues. TGF‐β1 is the key modulator of fibrogenesis in various tissues and the essential regulator in myofibroblast differentiation, leading to the apoptosis‐resistant phenotype by multiple signaling pathways. The suppression of Cav‐1 contributes to fibroblast proliferation and apoptosis resistance through TGF‐β1‐associated pathway in the development of idiopathic pulmonary fibrosis (IPF).^60^, ^61^, ^62^

However, other studies suggested that the downregulated Cav‐1 expression might reduce the severity of lung inflammation and vascular injury through activating polymorphonuclear neutrophils.^63^ The role of Cav‐1 in pulmonary arterial hypertension was verified in pulmonary arterial hypertension rat models, where Cav‐1 activated signal transducers and activators of transcription 3 (STAT3) transcription factor^64^ and regulated the bioavailability of NO.^65^ Increased Cav‐1 expression in pulmonary arterial hypertension enhanced agonist‐induced contraction via modulation of receptor‐operated calcium channels and store‐operated calcium channels in pulmonary arteries, playing a vital role in disease pathology.^66^ In lung cancer, Cav‐1 plays both suppressive and promoting roles.^67^ Downregulated Cav‐1 expression of cancer‐associated fibroblasts is observed in many aggressive cancers, indicating that Cav‐1 may inhibit tumor cell growth and increase the production of α‐smooth muscle actin, responsible for poor cancer outcomes.^68^ However, degradation of Cav‐1 can increase autophagy markers, such as cathepsin B (active form), lysosomal‐associated membrane protein‐1, LC3B, beclin 1, autophagy‐related 16 like 1, (ATG16L1), and BCL2 interacting protein 3 (BNIP3), to increase autophagy of cancer cells.^69^ Cav‐1 also regulates cellular senescence, for example, senescent lung fibroblasts, contributing to the progression of lung cancer.^70^, ^71^ Cav‐1 was identified to modulate the secretion of interleukin‐6 (IL‐6), which is an important factor in the microenvironment of tumor and the growth of cancer cells. Overexpression of Cav‐1 may induce premature senescence. Senescent fibroblasts stimulate the growth of cancer cells by secretion of IL‐6.^72^

### 1.3 Epigenetic modifications of Cav‐1 in healthy and diseased lungs

**FIGURE 3:**
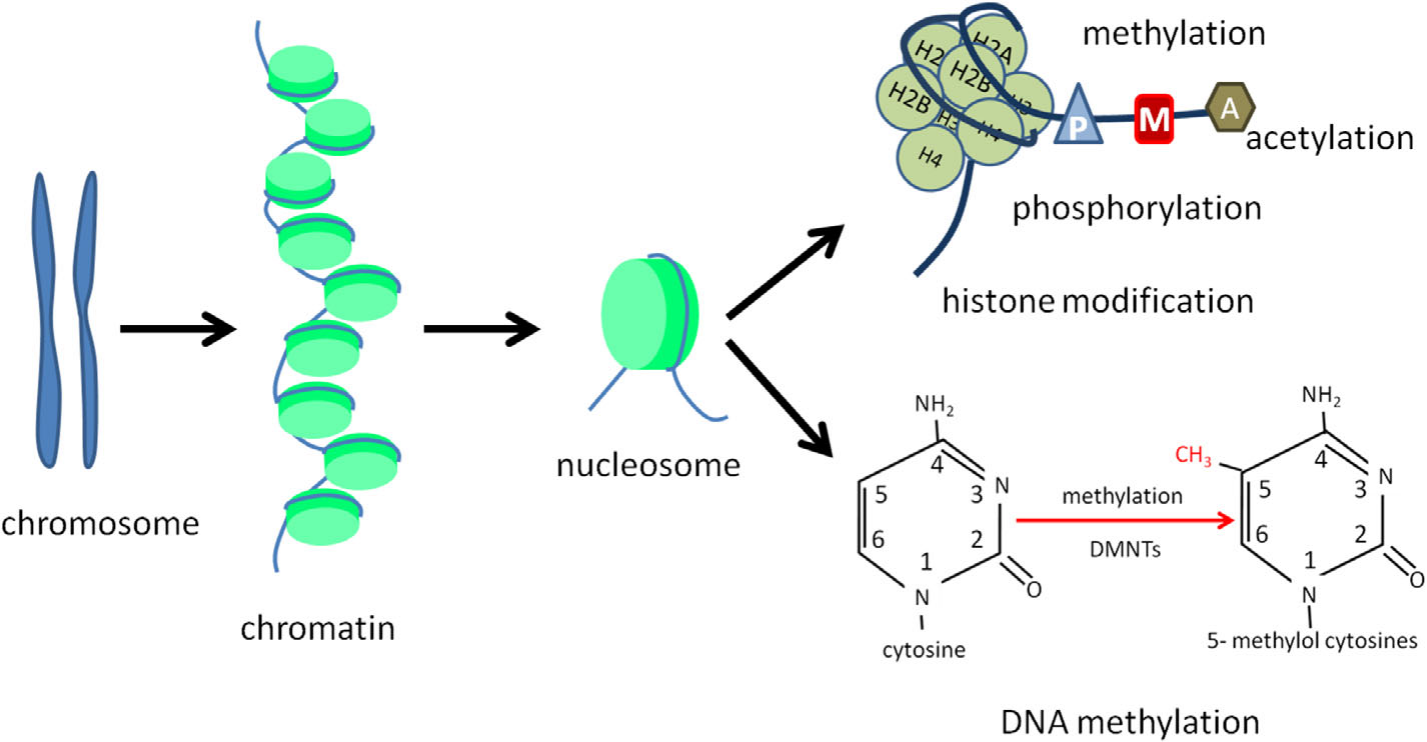
Two major epigenetics influence in gene expression. There are two major mechanisms involved in epigenetic regulation. One is the DNA methylation, changes on that could influence the gene expression level of gene. The other one is histone modification, which contains phosphorylation, methylation, and acetylation

**FIGURE 4:**
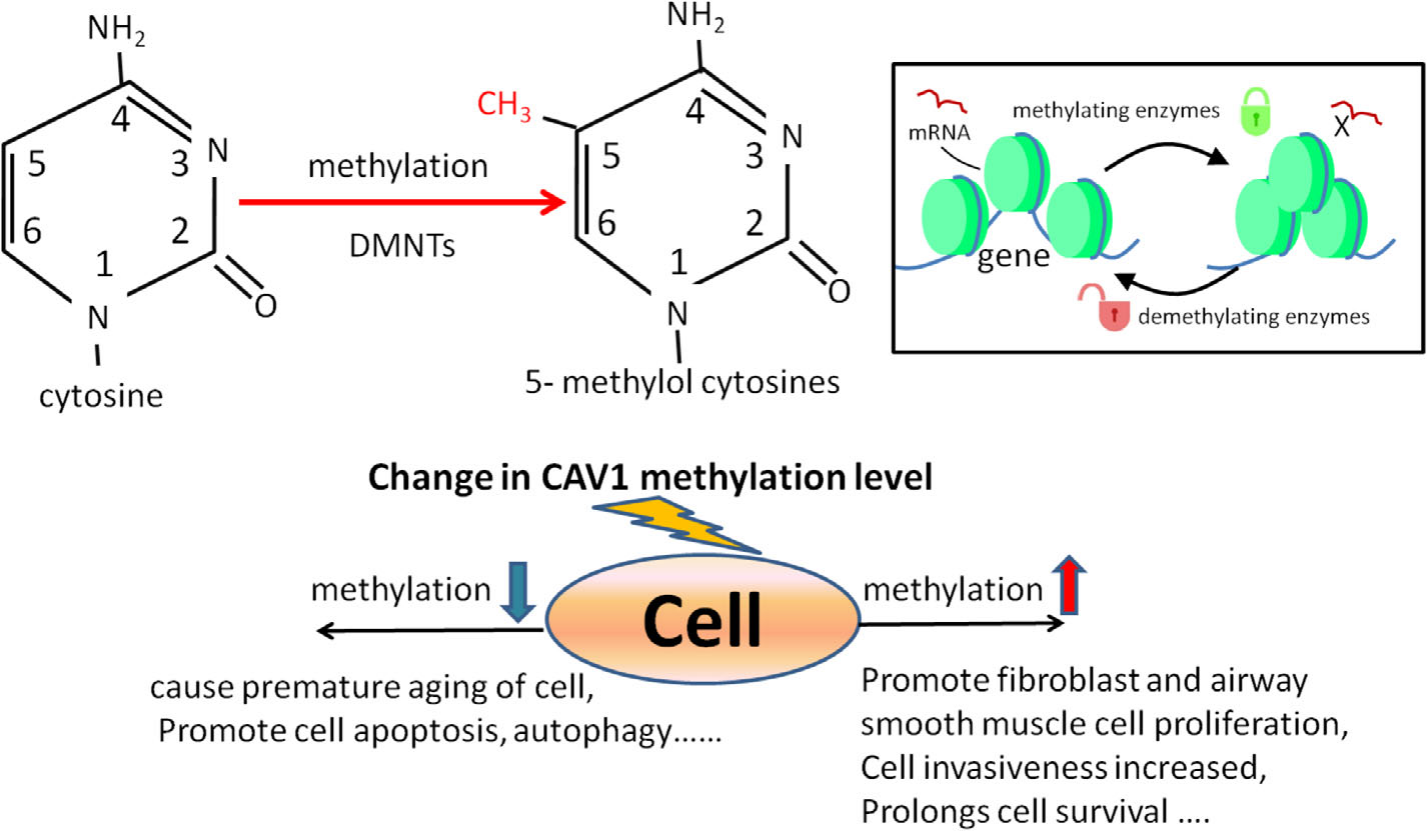
DNA methylation changes in *Cav*‐*1* gene expression. Methylation refers to the process of catalyzing the transfer of methyl groups from active methyl compounds to other compounds. DNA methylation generally occurs at the CpG site of gene promoter region. DNA methylation is an important modification of genes, which could regulate the expression level of genes and is closely related to many diseases. It is one of the crucial researches on epigenetic regulation

Epigenetics influence gene expression with changes in DNA sequences through two major mechanisms (Figure 3). Of those, DNA methylation involves methylation of gene promoter regions (Figure 4), whereas the histone modification is related to the structural changes of chromatin. Changes in epigenetic regulation can be restored by using some chemical agents.^73^ DNA promoter hypermethylation is induced by the modification of cytosine residues in the CpG dinucleotides to constitute 5‐methylcytosine via covalent addition of methyl group with DNA methyltransferase. CpG dinucleotides are disproportionally distributed in mammals. The CpG islands (CpGi) DNA within the gene promoters is a short sequence with high densities of CpG dinucleotides. The promoter region of genes with methylated CpGi is transcriptionally inactive due to the inhibitory role of methyl groups in transcriptional elements via accessing the promoter region.^73^, ^74^ The downregulation of Cav‐1 in various diseases is caused by the methylation at the *Cav‐1* coding genes CAV1 promoter region.

Cav‐1 can be downregulated by the aberrant promoter methylation of CAV1 in the stage and may be crucial in the development of many cancers.^75^, ^76^ The DNA methyltransferases play key roles in CAV1 expression in different stages of many cancers.^77^, ^78^ The positive or negative effects of Cav‐1 vary among a variety of aspects of tumor progression, due to the direct or indirect interaction of Cav‐1 with effector molecules to affect caveolae's function.^79^ The promoter CpG hypermethylation of CAV1 occurred at the onset of tumor development though a hypermethylated state remains in full‐blown tumors.^80^ However, the degree of methylation in metastatic foci and lymph nodes decreased to re‐express those related genes. Those genes are most partly inactivated through changes in DNA methylation and reactivated in demethylation activity.^81^ It was reported that the 5′ promoter of CAV1 was methylated in human breast cancer cells, whereas not in the normal human mammary epithelial cells.^82^ Furthermore, hypermethylation in CAV1 promoter region is involved in the histopathological grading of the tumor^83^ and with nodal metastasis, which is the most common form of metastasis pattern.^84^ Although there were different epigenetic changes in Cav‐1 among breast cancer subtypes, for example, CAV1 was overexpressed after being hypomethylated in inflammatory breast cancer.^85^ In addition to cancers, there are many other diseases involving the regulation of Cav‐1 methylation. Epigenetic regulation in Cav‐1 could protect cardiac function from ischaemic injury as a potential mechanism of cardioprotection.^86^ CAV1 deletion decreased expression of sirtuin1 in the ischemic preconditioning heart, which may affect DNA methylation across the genome and play a protective role in cardiac ischemia reperfusion injury.^86^

### 1.4 Caveolin‐1 methylation in chronic lung diseases

**FIGURE 5:**
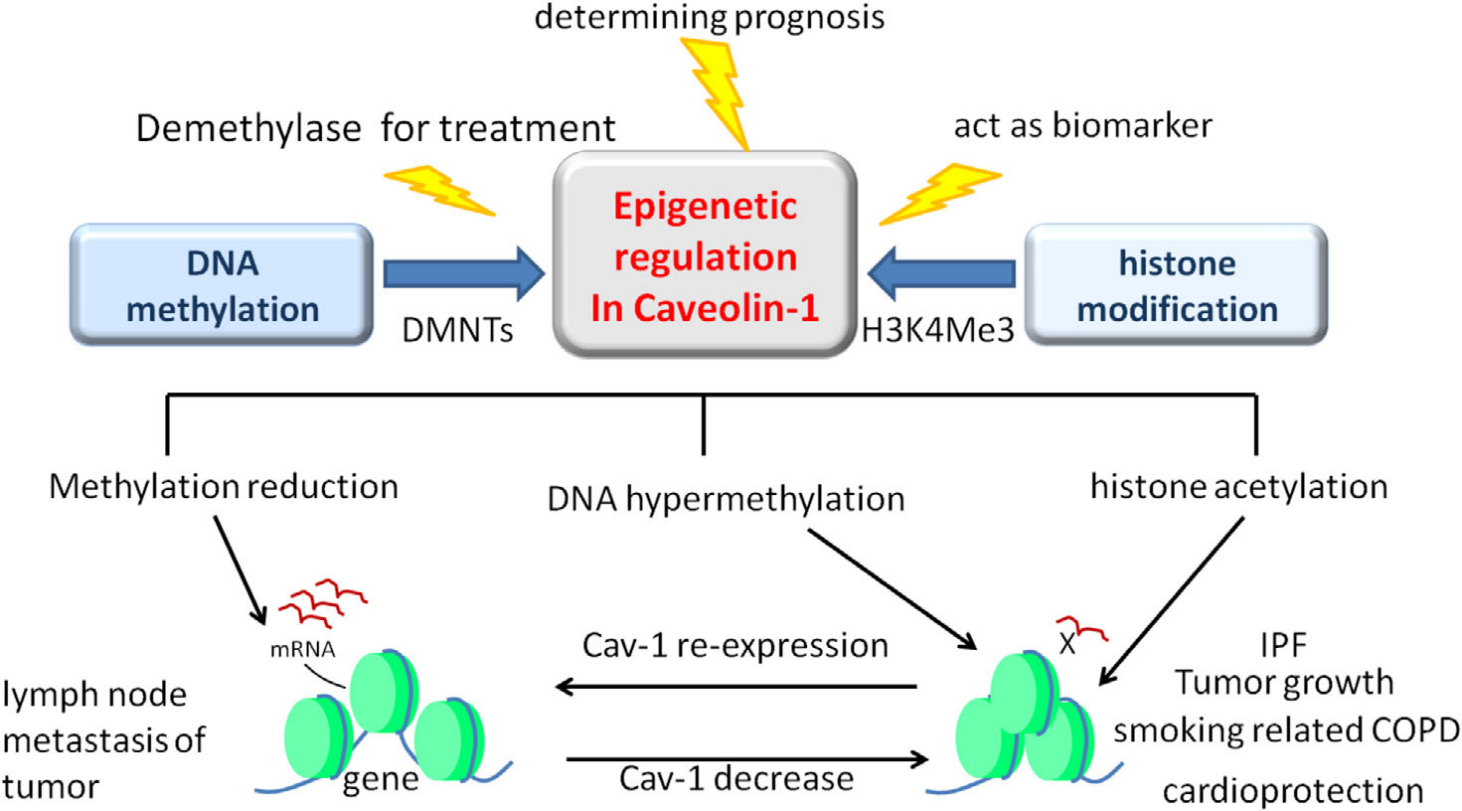
Epigenetic regulation changes in *Cav*‐*1* gene expression. DNA methylation and histone modification are the two major mechanisms of *Cav‐1* gene. Epigenetic regulation changes in *Cav‐1* gene could act as diagnosis and prognosis biomarkers of various lung diseases. Application of demethylase can reverse the methylation degree changes in promoter region of *Cav‐1* gene and induce the re‐expression of Cav‐1. The re‐expression of Cav‐1 makes therapeutic effect on some chronic lung diseases

The epigenetic changes in Cav‐1 may be a new target for the treatment of chronic lung diseases (Figure 5). Suppression of Cav‐1 expression was related to the gene promoter hypermethylated in COPD as well as in IPF.^87^ Compared with lung tissue in the COPD group and the nonsmoker group, the CpG sites of CAV1 in the COPD group were significantly hypermethylated.^88^ DNA methylation is seriously disrupted because of cigarette smoking, responsible for a wide range of malignant and nonmalignant diseases progression. It is an important mechanism contributing to COPD pathology. Abnormal CAV1 methylation was a whole genome phenomenon in small airways of patients with COPD, altering gene expressions and pathway activities important to COPD.^87^ Cav‐1 methylation can be a powerful predictor in the stable stage of lung cancer, and a potential biomarker for taxane‐based chemotherapy in lung cancers.^89^ *Cav‐1* gene methylation was related to overall survival of patients with lung cancer treated with taxane, although Cav‐1 expression levels did not show significant difference.^89^ Those effects of CAV1 promoter methylation in lung cancers seem to be cell and tissue specific. CAV1 could be a key molecule for lung cancer development. It plays quite different roles between small‐cell lung cancer and nonsmall‐cell lung cancer because the changes in CAV1 methylation may have opposite functions leading to either growth inhibition or growth promotion. For example, *CAV1* has been considered as a tumor suppressor gene in SCLC, whereas in NSCLC, *CAV1* acts as an oncogene and is responsible for survival and growth of tumor cells.^67^ Other epigenetic mechanisms, such as histone modifications, were observed in chronic lung diseases, for example, Cav‐1 expression was suppressed by the histone deacetylase inhibitor, trichostatin A.^90^ Expression of Cav‐1 was downregulated in IPF, when CAV1 was silenced through diminished binding of the active histone mark histone H3 trimethyl Lys4 with its promoter region.^91^ Combining with the evidences that Cav‐1 expression is significantly reduced in a variety of chronic lung diseases, we suspect that epigenetic changes of Cav‐1 may be a key pathological mechanism of chronic lung diseases.

The methylation of Cav‐1 promoter region by DNA methyltransferases is reversible and can be a new direction for targeted treatment of diseases. Treatment with a DNA methyltransferase inhibitor in breast cancer cell lines leads to the re‐expression of Cav‐1 through demethylation of CpGi shores.^92^ Treatment with 5‐AZA, which may reverse DNA promoter hypermethylation, could cause Cav‐1 re‐expression and restoration in ovarian cancer‐associated hypermethylation.^90^ Hypermethylation in Cav‐1 promoter region was reported in patients with colorectal cancer, whereas 5‐AZA could inhibit colon cancer cell growth through the Cav‐1 signal pathways.^93^, ^94^ Further, 5‐AZA treatment in hepatoma cells also leads to upregulated Cav‐1 expression.^95^ DNA is not easily degraded, DNA methylation happens uniquely in the CpG‐rich region and can be detected easily with a single pair of primers, or Cav‐1 can be secreted into the plasma and detected. Therefore, the treatment of abnormal methylated DNA by methyltransferase inhibitors is feasible, which can trigger the re‐expression of silenced genes, thereby improving the treatment efficiency.

## 2 CONCLUSIONS

In conclusion, Cav‐1 is important in healthy and diseased lungs, of which the suppression of Cav‐1 expression and function may be associated with the pathogenesis of chronic lung disease. Cav‐1, especially altered DNA methylation patterns in the promoter region, was associated with chronic lung diseases. Treatment with DNA methyltransferase inhibitor can activate Cav‐1 through demethylation of CpGi shores as therapeutic potentials for lung diseases, although there still are a large number of challenges to be overcome to meet criteria of disease‐specific biomarkers and targets to dynamically monitor disease severity, duration, stage, and response to therapy.^96^, ^97^, ^98^, ^99^, ^100^, ^101^, ^102^, ^103^, ^104^, ^105^, ^106^, ^107^, ^108^, ^109^, ^110^, ^111^ Understanding of Cav‐1 may contribute to developing the new therapies. Further researches will be needed to clarify the role of CAV1 in the development of chronic lung disease and to determine whether CAV1 expression and/or promoter methylation could be used as an alternative of diagnostic biomarkers and therapeutic targets for chronic lung diseases in the early diagnosis and clinical treatment.

## CONFLICT OF INTEREST

The authors declare that they have no conflict of interest.

## References

[1] Cohen AW , Hnasko R , Schubert W , Lisanti MP , Role of caveolae and caveolins in health and disease. Physiol Rev. 2004;84:1341‐1379. 15383654 10.1152/physrev.00046.2003

[2] Razani B , Wang XB , Engelman JA , etal . Caveolin‐2‐deficient mice show evidence of severe pulmonary dysfunction without disruption of caveolae. Mol Cell Biol. 2002;22:2329‐2344. 11884617 10.1128/MCB.22.7.2329-2344.2002 PMC133690

[3] Galbiati F , Engelman JA , Volonte D , et al. Caveolin‐3 null mice show a loss of caveolae, changes in the microdomain distribution of the dystrophin‐glycoprotein complex, and t‐tubule abnormalities. J Biol Chem. 2001;276:21425‐21433. 11259414 10.1074/jbc.M100828200

[4] Ariotti N , Rae J , Leneva N , etal . Molecular characterization of caveolin‐induced membrane curvature. J Biol Chem. 2015;290:24875‐24890. 26304117 10.1074/jbc.M115.644336 PMC4598997

[5] Whiteley G , Collins RF , Kitmitto A , Characterization of the molecular architecture of human caveolin‐3 and interaction with the skeletal muscle ryanodine receptor. J Biol Chem. 2012;287:40302‐40316. 23071107 10.1074/jbc.M112.377085 PMC3504746

[6] Liu H , Yang L , Zhang Q , Mao L , Jiang H , Yang H , Probing the structure and dynamics of caveolin‐1 in a caveolae‐mimicking asymmetric lipid bilayer model. Eur Biophys J. 2016;45:511‐521. 27038819 10.1007/s00249-016-1118-1

[7] Atshaves BP , Jefferson JR , McIntosh AL , etal . Effect of sterol carrier protein‐2 expression on sphingolipid distribution in plasma membrane lipid rafts/caveolae. Lipids. 2007;42:871‐884. 17680294 10.1007/s11745-007-3091-z

[8] Martinez‐Outschoorn UE , Sotgia F , Lisanti MP , Caveolae and signalling in cancer. Nat Rev Cancer. 2015;15:225‐237. 25801618 10.1038/nrc3915

[9] Epand RM , Sayer BG , Epand RF , Caveolin scaffolding region and cholesterol‐rich domains in membranes. J Mol Biol. 2005;345:339‐350. 15571726 10.1016/j.jmb.2004.10.064

[10] Sohn J , Lin H , Fritch MR , Tuan RS , Influence of cholesterol/caveolin‐1/caveolae homeostasis on membrane properties and substrate adhesion characteristics of adult human mesenchymal stem cells. Stem Cell Res Ther. 2018;9:86. 29615119 10.1186/s13287-018-0830-4 PMC5883280

[11] Razani B , Engelman JA , Wang XB , et al. Caveolin‐1 null mice are viable but show evidence of hyperproliferative and vascular abnormalities. J Biol Chem. 2001;276:38121‐38138. 11457855 10.1074/jbc.M105408200

[12] Drab M , Verkade P , Elger M , et al. Loss of caveolae, vascular dysfunction, and pulmonary defects in caveolin‐1 gene‐disrupted mice. Science. 2001;293:2449‐2452. 11498544 10.1126/science.1062688

[13] Yang G , Dong Z , Xu H , et al. Structural study of caveolin‐1 intramembrane domain by circular dichroism and nuclear magnetic resonance. Biopolymers. 2015;104:11‐20. 25471446 10.1002/bip.22597

[14] Nohe A , Keating E , Loh C , Underhill MT , Petersen NO , Caveolin‐1 isoform reorganization studied by image correlation spectroscopy. Faraday Discuss. 2004;126:185‐195; discussion 245‐254. 14992406 10.1039/b304943d

[15] Kogo H , Aiba T , Fujimoto T , Cell type‐specific occurrence of caveolin‐1alpha and ‐1beta in the lung caused by expression of distinct mRNAs. J Biol Chem. 2004;279:25574‐25581. 15067006 10.1074/jbc.M310807200

[16] Kogo H , Fujimoto T , Caveolin‐1 isoforms are encoded by distinct mRNAs. Identification of mouse caveolin‐1 mRNA variants caused by alternative transcription initiation and splicing. FEBS Lett. 2000;465:119‐123. 10631317 10.1016/s0014-5793(99)01730-5

[17] Ramirez MI , Pollack L , Millien G , Cao YX , Hinds A , Williams MC , The alpha‐isoform of caveolin‐1 is a marker of vasculogenesis in early lung development. J Histochem Cytochem. 2002;50:33‐42. 11748292 10.1177/002215540205000104

[18] Nohe A , Keating E , Underhill TM , Knaus P , Petersen NO , Dynamics and interaction of caveolin‐1 isoforms with BMP‐receptors. J Cell Sci. 2005;118:643‐650. 15657086 10.1242/jcs.01402

[19] Fujimoto T , Kogo H , Nomura R , Une T , Isoforms of caveolin‐1 and caveolar structure. J Cell Sci. 2000;113(Pt 19):3509‐3517. 10984441 10.1242/jcs.113.19.3509

[20] Fairn GD , Schieber NL , Ariotti N , et al. High‐resolution mapping reveals topologically distinct cellular pools of phosphatidylserine. J Cell Biol. 2011;194:257‐275. 21788369 10.1083/jcb.201012028 PMC3144401

[21] Goetz JG , Joshi B , Lajoie P , et al. Concerted regulation of focal adhesion dynamics by galectin‐3 and tyrosine‐phosphorylated caveolin‐1. J Cell Biol. 2008;180:1261‐1275. 18347068 10.1083/jcb.200709019 PMC2290850

[22] Wang XM , Zhang Y , Kim HP , et al. Caveolin‐1: a critical regulator of lung fibrosis in idiopathic pulmonary fibrosis. J Exp Med. 2006;203:2895‐2906. 17178917 10.1084/jem.20061536 PMC1850940

[23] Chen Z , Bakhshi FR , Shajahan AN , et al. Nitric oxide‐dependent Src activation and resultant caveolin‐1 phosphorylation promote eNOS/caveolin‐1 binding and eNOS inhibition. Mol Biol Cell. 2012;23:1388‐1398. 22323292 10.1091/mbc.E11-09-0811 PMC3315804

[24] Minguet S , Klasener K , Schaffer AM , et al. Caveolin‐1‐dependent nanoscale organization of the BCR regulates B cell tolerance. Nat Immunol. 2017;18:1150‐1159. 28805811 10.1038/ni.3813 PMC5608079

[25] Sun LN , Chen ZX , Liu XC , Liu HY , Guan GJ , Liu G , Curcumin ameliorates epithelial‐to‐mesenchymal transition of podocytes in vivo and in vitro via regulating caveolin‐1. Biomed Pharmacother. 2014;68:1079‐1088. 25456852 10.1016/j.biopha.2014.10.005

[26] Wang N , Zhang D , Sun G , et al. Lipopolysaccharide‐induced caveolin‐1 phosphorylation‐dependent increase in transcellular permeability precedes the increase in paracellular permeability. Drug Des Devel Ther. 2015;9:4965‐4977. 10.2147/DDDT.S77646 PMC4560510 26357463

[27] Patel HH , Murray F , Insel PA , Caveolae as organizers of pharmacologically relevant signal transduction molecules. Annu Rev Pharmacol Toxicol. 2008;48:359‐391. 17914930 10.1146/annurev.pharmtox.48.121506.124841 PMC3083858

[28] Kang Q , Xiang Y , Li D , et al. MiR‐124‐3p attenuates hyperphosphorylation of Tau protein‐induced apoptosis via caveolin‐1‐PI3K/Akt/GSK3beta pathway in N2a/APP695swe cells. Oncotarget. 2017;8:24314‐24326. 28186985 10.18632/oncotarget.15149 PMC5421849

[29] Samarakoon R , Chitnis SS , Higgins SP , et al. Redox‐induced Src kinase and caveolin‐1 signaling in TGF‐beta1‐initiated SMAD2/3 activation and PAI‐1 expression. PLoS One. 2011;6:e22896. 21829547 10.1371/journal.pone.0022896 PMC3145778

[30] Diaz J , Mendoza P , Ortiz R , et al. Rab5 is required in metastatic cancer cells for caveolin‐1‐enhanced Rac1 activation, migration and invasion. J Cell Sci. 2014;127:2401‐2406. 24659799 10.1242/jcs.141689 PMC4074264

[31] Chen Z , S DSO , Zimnicka AM , et al. Reciprocal regulation of eNOS and caveolin‐1 functions in endothelial cells. Mol Biol Cell. 2018;29:1190‐1202. 29563255 10.1091/mbc.E17-01-0049 PMC5935069

[32] Klinz FJ , Schmidt A , Schinkothe T , et al. Phospho‐eNOS Ser‐114 in human mesenchymal stem cells: constitutive phosphorylation, nuclear localization and upregulation during mitosis. Eur J Cell Biol. 2005;84:809‐818. 16270749 10.1016/j.ejcb.2005.06.003

[33] Komers R , Schutzer WE , Reed JF , et al. Altered endothelial nitric oxide synthase targeting and conformation and caveolin‐1 expression in the diabetic kidney. Diabetes. 2006;55:1651‐1659. 16731827 10.2337/db05-1595

[34] Fujita Y , Maruyama S , Kogo H , Matsuo S , Fujimoto T , Caveolin‐1 in mesangial cells suppresses MAP kinase activation and cell proliferation induced by bFGF and PDGF. Kidney Int. 2004;66:1794‐1804. 15496150 10.1111/j.1523-1755.2004.00954.x

[35] Gosens R , Dueck G , Gerthoffer WT , et al. p42/p44 MAP kinase activation is localized to caveolae‐free membrane domains in airway smooth muscle. Am J Physiol Lung Cell Mol Physiol. 2007;292:L1163‐L1172. 17237147 10.1152/ajplung.00471.2006

[36] Cohen AW , Park DS , Woodman SE , et al. Caveolin‐1 null mice develop cardiac hypertrophy with hyperactivation of p42/44 MAP kinase in cardiac fibroblasts. Am J Physiol Cell Physiol. 2003;284:C457‐C474. 12388077 10.1152/ajpcell.00380.2002

[37] Bosch M , Mari M , Herms A , et al. Caveolin‐1 deficiency causes cholesterol‐dependent mitochondrial dysfunction and apoptotic susceptibility. Curr Biol. 2011;21:681‐686. 21497090 10.1016/j.cub.2011.03.030 PMC3409647

[38] Fernandez‐Rojo MA , Gongora M , Fitzsimmons RL , et al. Caveolin‐1 is necessary for hepatic oxidative lipid metabolism: evidence for crosstalk between caveolin‐1 and bile acid signaling. Cell Rep. 2013;4:238‐247. 23850288 10.1016/j.celrep.2013.06.017

[39] Kawaraguchi Y , Horikawa YT , Murphy AN , et al. Volatile anesthetics protect cancer cells against tumor necrosis factor‐related apoptosis‐inducing ligand‐induced apoptosis via caveolins. Anesthesiology. 2011;115:499‐508. 21862885 10.1097/ALN.0b013e3182276d42 PMC3162469

[40] Ryter SW , Lam HC , Chen ZH , Choi AM , Deadly triplex: smoke, autophagy and apoptosis. Autophagy. 2011;7:436‐437. 21200154 10.4161/auto.7.4.14501

[41] Williams TM , Lisanti MP , The caveolin proteins. Genome Biol. 2004;5:214. 15003112 10.1186/gb-2004-5-3-214 PMC395759

[42] Schubert W , Frank PG , Woodman SE , et al. Microvascular hyperpermeability in caveolin‐1 (‐/‐) knock‐out mice. Treatment with a specific nitric‐oxide synthase inhibitor, L‐NAME, restores normal microvascular permeability in Cav‐1 null mice. J Biol Chem. 2002;277:40091‐40098. 12167625 10.1074/jbc.M205948200

[43] Razani B , Combs TP , Wang XB , et al. Caveolin‐1‐deficient mice are lean, resistant to diet‐induced obesity, and show hypertriglyceridemia with adipocyte abnormalities. J Biol Chem. 2002;277:8635‐8647. 11739396 10.1074/jbc.M110970200

[44] Asterholm IW , Mundy DI , Weng J , Anderson RG , Scherer PE , Altered mitochondrial function and metabolic inflexibility associated with loss of caveolin‐1. Cell Metab. 2012;15:171‐185. 22326219 10.1016/j.cmet.2012.01.004 PMC3278712

[45] Kim CA , Delepine M , Boutet E , et al. Association of a homozygous nonsense caveolin‐1 mutation with Berardinelli‐Seip congenital lipodystrophy. J Clin Endocrinol Metab. 2008;93:1129‐1134. 18211975 10.1210/jc.2007-1328

[46] Gosens R , Mutawe M , Martin S , et al. Caveolae and caveolins in the respiratory system. Curr Mol Med. 2008;8:741‐753. 19075672 10.2174/156652408786733720

[47] Royce SG , Le Saux CJ , Role of caveolin‐1 in asthma and chronic inflammatory respiratory diseases. Expert Rev Respir Med. 2014;8:339‐347. 24742020 10.1586/17476348.2014.905915

[48] Maniatis NA , Chernaya O , Shinin V , Minshall RD , Caveolins and lung function. Adv Exp Med Biol. 2012;729:157‐179. 22411320 10.1007/978-1-4614-1222-9_11 PMC3449096

[49] Gvaramia D , Blaauboer ME , Hanemaaijer R , Everts V , Role of caveolin‐1 in fibrotic diseases. Matrix Biol. 2013;32:307‐315. 23583521 10.1016/j.matbio.2013.03.005

[50] Ji DG , Zhang Y , Yao SM , et al. Cav‐1 deficiency promotes liver fibrosis in carbon tetrachloride (CCl4)‐induced mice by regulation of oxidative stress and inflammation responses. Biomed Pharmacother. 2018;102:26‐33. 29549726 10.1016/j.biopha.2018.03.016

[51] Murata T , Lin MI , Huang Y , et al. Reexpression of caveolin‐1 in endothelium rescues the vascular, cardiac, and pulmonary defects in global caveolin‐1 knockout mice. J Exp Med. 2007;204:2373‐2382. 17893196 10.1084/jem.20062340 PMC2118452

[52] Maniatis NA , Balyasnikova IV , Metzger R , et al. Reduced expression of angiotensin I‐converting enzyme in caveolin‐1 knockout mouse lungs. Microvasc Res. 2010;80:250‐257. 20430040 10.1016/j.mvr.2010.04.008 PMC2919634

[53] Gabehart KE , Royce SG , Maselli DJ , et al. Airway hyperresponsiveness is associated with airway remodeling but not inflammation in aging Cav1‐/‐ mice. Respir Res. 2013;14:110. 24138138 10.1186/1465-9921-14-110 PMC4015038

[54] Volonte D , Kahkonen B , Shapiro S , Di Y , Galbiati F , Caveolin‐1 expression is required for the development of pulmonary emphysema through activation of the ATM‐p53‐p21 pathway. J Biol Chem. 2009;284:5462‐5466. 19103597 10.1074/jbc.C800225200 PMC2645811

[55] Sun N , Wei X , Wang J , Cheng Z , Sun W , Caveolin‐1 promotes the imbalance of Th17/Treg in patients with chronic obstructive pulmonary disease. Inflammation. 2016;39:2008‐2015. 27613621 10.1007/s10753-016-0436-x

[56] Garrean S , Gao XP , Brovkovych V , et al. Caveolin‐1 regulates NF‐kappaB activation and lung inflammatory response to sepsis induced by lipopolysaccharide. J Immunol. 2006;177:4853‐4860. 16982927 10.4049/jimmunol.177.7.4853

[57] Jin Y , Lee SJ , Minshall RD , Choi AM , Caveolin‐1: a critical regulator of lung injury. Am J Physiol Lung Cell Mol Physiol. 2011;300:L151‐L160. 21097526 10.1152/ajplung.00170.2010 PMC4380484

[58] Bains SN , Tourkina E , Atkinson C , et al. Loss of caveolin‐1 from bronchial epithelial cells and monocytes in human subjects with asthma. Allergy. 2012;67:1601‐1604. 23004679 10.1111/all.12021 PMC3499648

[59] Wang KY , Lee MF , Ho HC , et al. Serum caveolin‐1 as a novel biomarker in idiopathic pulmonary artery hypertension. Biomed Res Int. 2015;2015:173970. 26539466 10.1155/2015/173970 PMC4619756

[60] Darby IA , Hewitson TD , Fibroblast differentiation in wound healing and fibrosis. Int Rev Cytol. 2007;257:143‐179. 17280897 10.1016/S0074-7696(07)57004-X

[61] Xia H , Khalil W , Kahm J , Jessurun J , Kleidon J , Henke CA , Pathologic caveolin‐1 regulation of PTEN in idiopathic pulmonary fibrosis. Am J Pathol. 2010;176:2626‐2637. 20395445 10.2353/ajpath.2010.091117 PMC2877826

[62] Cerezo A , Guadamillas MC , Goetz JG , et al. The absence of caveolin‐1 increases proliferation and anchorage‐independent growth by a Rac‐dependent, Erk‐independent mechanism. Mol Cell Biol. 2009;29:5046‐5059. 19620284 10.1128/MCB.00315-09 PMC2738299

[63] Lv XJ , Li YY , Zhang YJ , Mao M , Qian GS , Over‐expression of caveolin‐1 aggravate LPS‐induced inflammatory response in AT‐1 cells via up‐regulation of cPLA2/p38 MAPK. Inflamm Res. 2010;59:531‐541. 20099006 10.1007/s00011-010-0157-9

[64] Mathew R , Huang J , Shah M , Patel K , Gewitz M , Sehgal PB , Disruption of endothelial‐cell caveolin‐1alpha/raft scaffolding during development of monocrotaline‐induced pulmonary hypertension. Circulation. 2004;110:1499‐1506. 15353500 10.1161/01.CIR.0000141576.39579.23

[65] Mukhopadhyay S , Xu F , Sehgal PB , Aberrant cytoplasmic sequestration of eNOS in endothelial cells after monocrotaline, hypoxia, and senescence: live‐cell caveolar and cytoplasmic NO imaging. Am J Physiol Heart Circ Physiol. 2007;292:H1373‐H1389. 17071725 10.1152/ajpheart.00990.2006

[66] Jiao HX , Mu YP , Gui LX , et al. Increase in caveolae and caveolin‐1 expression modulates agonist‐induced contraction and store‐ and receptor‐operated Ca(2+) entry in pulmonary arteries of pulmonary hypertensive rats. Vascul Pharmacol. 2016;84:55‐66. 27311393 10.1016/j.vph.2016.06.004

[67] Sunaga N , Miyajima K , Suzuki M , et al. Different roles for caveolin‐1 in the development of non‐small cell lung cancer versus small cell lung cancer. Cancer Res. 2004;64:4277‐4285. 15205342 10.1158/0008-5472.CAN-03-3941

[68] Fiering S , Ang LH , Lacoste J , Smith TD , Griner E , Registered report: biomechanical remodeling of the microenvironment by stromal caveolin‐1 favors tumor invasion and metastasis. Elife. 2015;4:e04796. 26179155 10.7554/eLife.04796 PMC4503935

[69] Martinez‐Outschoorn UE , Trimmer C , Lin Z , et al. Autophagy in cancer associated fibroblasts promotes tumor cell survival: role of hypoxia, HIF1 induction and NFkappaB activation in the tumor stromal microenvironment. Cell Cycle. 2010;9:3515‐3533. 20855962 10.4161/cc.9.17.12928 PMC3047617

[70] Bartholomew JN , Volonte D , Galbiati F , Caveolin‐1 regulates the antagonistic pleiotropic properties of cellular senescence through a novel Mdm2/p53‐mediated pathway. Cancer Res. 2009;69:2878‐2886. 19318577 10.1158/0008-5472.CAN-08-2857 PMC2692066

[71] Papadopoulou A , Kletsas D , Human lung fibroblasts prematurely senescent after exposure to ionizing radiation enhance the growth of malignant lung epithelial cells in vitro and in vivo. Int J Oncol. 2011;39:989‐999. 21814715 10.3892/ijo.2011.1132

[72] Volonte D , Zou H , Bartholomew JN , Liu Z , Morel PA , Galbiati F , Oxidative stress‐induced inhibition of Sirt1 by caveolin‐1 promotes p53‐dependent premature senescence and stimulates the secretion of interleukin 6 (IL‐6). J Biol Chem. 2015;290:4202‐4214. 25512378 10.1074/jbc.M114.598268 PMC4326829

[73] Verma M , Srivastava S , Epigenetics in cancer: implications for early detection and prevention. Lancet Oncol. 2002;3:755‐763. 12473517 10.1016/s1470-2045(02)00932-4

[74] De Carvalho DD , You JS , Jones PA , DNA methylation and cellular reprogramming. Trends Cell Biol. 2010;20:609‐617. 20810283 10.1016/j.tcb.2010.08.003 PMC2981432

[75] Lin SY , Yeh KT , Chen WT , Chen HC , Chen ST , Chang JG , Promoter CpG methylation of caveolin‐1 in sporadic colorectal cancer. Anticancer Res. 2004;24:1645‐1650. 15274335

[76] Chen ST , Lin SY , Yeh KT , et al. Mutational, epigenetic and expressional analyses of caveolin‐1 gene in breast cancers. Int J Mol Med. 2004;14:577‐582. 15375584

[77] Deb M , Sengupta D , Kar S , et al. Elucidation of caveolin 1 both as a tumor suppressor and metastasis promoter in light of epigenetic modulators. Tumour Biol. 2014;35:12031‐12047. 25192721 10.1007/s13277-014-2502-z

[78] Sundar IK , Mullapudi N , Yao H , Spivack SD , Rahman I , Lung cancer and its association with chronic obstructive pulmonary disease: update on nexus of epigenetics. Curr Opin Pulm Med. 2011;17:279‐285. 21537190 10.1097/MCP.0b013e3283477533 PMC3730439

[79] Goetz JG , Lajoie P , Wiseman SM , Nabi IR , Caveolin‐1 in tumor progression: the good, the bad and the ugly. Cancer Metastasis Rev. 2008;27:715‐735. 18506396 10.1007/s10555-008-9160-9

[80] Patra SK , Bettuzzi S , Epigenetic DNA‐methylation regulation of genes coding for lipid raft‐associated components: a role for raft proteins in cell transformation and cancer progression (review). Oncol Rep. 2007;17:1279‐1290. 17487380

[81] Patra SK , Dissecting lipid raft facilitated cell signaling pathways in cancer. Biochim Biophys Acta. 2008;1785:182‐206. 18166162 10.1016/j.bbcan.2007.11.002

[82] Li Z , Guo X , Wu Y , et al. Methylation profiling of 48 candidate genes in tumor and matched normal tissues from breast cancer patients. Breast Cancer Res Treat. 2015;149:767‐779. 25636590 10.1007/s10549-015-3276-8

[83] Syeed N , Hussain F , Husain SA , Siddiqi MA , 5′‐CpG island promoter hypermethylation of the *CAV*‐1 gene in breast cancer patients of Kashmir. Asian Pac J Cancer Prev. 2012;13:371‐375. 22502704 10.7314/apjcp.2012.13.1.371

[84] Alevizos L , Kataki A , Derventzi A , et al. Breast cancer nodal metastasis correlates with tumour and lymph node methylation profiles of caveolin‐1 and CXCR4. Clin Exp Metastasis. 2014;31:511‐520. 24590865 10.1007/s10585-014-9645-6

[85] Van den Eynden GG , Van Laere SJ , Van der Auwera I , et al. Overexpression of caveolin‐1 and ‐2 in cell lines and in human samples of inflammatory breast cancer. Breast Cancer Res Treat. 2006;95:219‐228. 16244790 10.1007/s10549-005-9002-1

[86] Das M , Das S , Lekli I , Das DK , Caveolin induces cardioprotection through epigenetic regulation. J Cell Mol Med. 2012;16:888‐895. 21707918 10.1111/j.1582-4934.2011.01372.x PMC3822857

[87] Vucic EA , Chari R , Thu KL , et al. DNA methylation is globally disrupted and associated with expression changes in chronic obstructive pulmonary disease small airways. Am J Respir Cell Mol Biol. 2014;50:912‐922. 24298892 10.1165/rcmb.2013-0304OC PMC4068945

[88] Sundar IK , Yin Q , Baier BS , et al. DNA methylation profiling in peripheral lung tissues of smokers and patients with COPD. Clin Epigenetics. 2017;9:38. 28416970 10.1186/s13148-017-0335-5 PMC5391602

[89] Brodie SA , Lombardo C , Li G , et al. Aberrant promoter methylation of caveolin‐1 is associated with favorable response to taxane‐platinum combination chemotherapy in advanced NSCLC. PLoS One. 2014;9:e107124. 25222296 10.1371/journal.pone.0107124 PMC4164573

[90] Wiechen K , Diatchenko L , Agoulnik A , et al. Caveolin‐1 is down‐regulated in human ovarian carcinoma and acts as a candidate tumor suppressor gene. Am J Pathol. 2001;159:1635‐1643. 11696424 10.1016/S0002-9440(10)63010-6 PMC1867061

[91] Sanders YY , Liu H , Scruggs AM , Duncan SR , Huang SK , Thannickal VJ , Epigenetic regulation of caveolin‐1 gene expression in lung fibroblasts. Am J Respir Cell Mol Biol. 2017;56:50‐61. 27560128 10.1165/rcmb.2016-0034OC PMC5248956

[92] Rao X , Evans J , Chae H , et al. CpG island shore methylation regulates caveolin‐1 expression in breast cancer. Oncogene. 2013;32:4519‐4528. 23128390 10.1038/onc.2012.474 PMC3787796

[93] Mori Y , Cai K , Cheng Y , et al. A genome‐wide search identifies epigenetic silencing of somatostatin, tachykinin‐1, and 5 other genes in colon cancer. Gastroenterology. 2006;131:797‐808. 16952549 10.1053/j.gastro.2006.06.006

[94] Ha TK , Her NG , Lee MG , et al. Caveolin‐1 increases aerobic glycolysis in colorectal cancers by stimulating HMGA1‐mediated GLUT3 transcription. Cancer Res. 2012;72:4097‐4109. 22706202 10.1158/0008-5472.CAN-12-0448

[95] Hirasawa Y , Arai M , Imazeki F , et al. Methylation status of genes upregulated by demethylating agent 5‐aza‐2'‐deoxycytidine in hepatocellular carcinoma. Oncology. 2006;71:77‐85. 17341888 10.1159/000100475

[96] Shi L , Zhu B , Xu M , Wang X , Selection of AECOPD‐specific immunomodulatory biomarkers by integrating genomics and proteomics with clinical informatics. Cell Biol Toxicol. 2018;34:109‐123. 28779230 10.1007/s10565-017-9405-x

[97] Ansari D , Toren W , Zhou Q , Hu D , Andersson R , Proteomic and genomic profiling of pancreatic cancer. Cell Biol Toxicol. 2019;35:333‐343. 10.1007/s10565-019-09465-9 PMC6757097 30771135

[98] Marcell Szasz A , Malm J , Rezeli M , et al. Challenging the heterogeneity of disease presentation in malignant melanoma‐impact on patient treatment. Cell Biol Toxicol. 2019;35:1‐14. 30357519 10.1007/s10565-018-9446-9 PMC6514062

[99] Zhang L , Han X , Wang X , Is the clinical lipidomics a potential goldmine? Cell Biol Toxicol. 2018;34:421‐423. 30032454 10.1007/s10565-018-9441-1 PMC6208904

[100] Kawamura Y , Takouda J , Yoshimoto K , Nakashima K , New aspects of glioblastoma multiforme revealed by similarities between neural and glioblastoma stem cells. Cell Biol Toxicol. 2018;34:425‐440. 29383547 10.1007/s10565-017-9420-y

[101] Song D , Yang D , Powell CA , Wang X , Cell‐cell communication: old mystery and new opportunity. Cell Biol Toxicol. 2019;35:89‐93. 30815784 10.1007/s10565-019-09470-y

[102] Wang X , Clinical trans‐omics: an integration of clinical phenomes with molecular multiomics. Cell Biol Toxicol. 2018;34:163‐166. 29691682 10.1007/s10565-018-9431-3

[103] Wu D , Wang X , Sun H , The role of mitochondria in cellular toxicity as a potential drug target. Cell Biol Toxicol. 2018;34:87‐91. 29511917 10.1007/s10565-018-9425-1

[104] Zhu Z , Qiu S , Shao K , Hou Y , Progress and challenges of sequencing and analyzing circulating tumor cells. Cell Biol Toxicol. 2018;34:405‐415. 29168077 10.1007/s10565-017-9418-5 PMC6132989

[105] Wu D , Cheng Y , Wang XGroupCSGT , Definition of clinical gene tests. Cell Biol Toxicol. 2019;35:83‐87. 30746600 10.1007/s10565-019-09464-w

[106] Liu X , Wu J , History, applications, and challenges of immune repertoire research. Cell Biol Toxicol. 2018;34:441‐457. 29484527 10.1007/s10565-018-9426-0

[107] Um J , Jung DW , Williams DR , The future is now: cutting edge science and understanding toxicology. Cell Biol Toxicol. 2018;34:79‐85. 29397478 10.1007/s10565-018-9421-5

[108] Wang W , Gao D , Wang X , Can single‐cell RNA sequencing crack the mystery of cells? Cell Biol Toxicol. 2018;34:1‐6. 28733864 10.1007/s10565-017-9404-y

[109] Li R , Liu Y , Hou Y , Gan J , Wu P , Li C , 3D genome and its disorganization in diseases. Cell Biol Toxicol. 2018;34:351‐365. 29796744 10.1007/s10565-018-9430-4

[110] Zeng Y , Chen X , Gao H , Wang X , An artificial intelligent single cell is part of the cell dream world. Cell Biol Toxicol. 2018;34:247‐249. 29777423 10.1007/s10565-018-9433-1 PMC6021470

[111] Shi L , Dong N , Ji D , et al. Lipopolysaccharide‐induced CCN1 production enhances interleukin‐6 secretion in bronchial epithelial cells. Cell Biol Toxicol. 2018;34:39‐49. 28638955 10.1007/s10565-017-9401-1 PMC5775366

